# Cow's Milk and Rickets

**Published:** 1908-05-23

**Authors:** 


### EDITOR'S LETTER-BOX.

### COW'S MILK AND RICKETS.

Sir,—Let Dr. Stephens consider the following facts, or rather jokes. Text-books state that the proportion of salts (calcium phosphate being put first) in cow's milk is to that in human milk as 7 to 3. Presumably, then, one may dilute the former a little more than half without running the risk of giving the baby not enough lime. Practical people think that one may go further. In an address delivered at Great Ormond Street Hospital, and published in the *Lancet*, 1904, Dr. Poynton (who seems to think better of Sir A. E. Wright's work than Dr. Stephens does) describes a plan of diluting milk with an equal part of water, and then decalcifying it with one or even two grains of sodium citrate to the ounce. He says that there is no danger of rickets, and that he has never seen any failure in bone formation. But Dr. Stephens states that the usual dilution may go as far as 1 in 5. Goodhart and Still are not of this opinion, and only recommend 1 in 4 during the first week of infant life, a period surely too short for much harm to be done; and they note the well-known fact that some infants thrive quite well on pure cow's milk, which a fair number of doctors will tell one they recommend. Surely these babies are getting enough calcium in their milk.

The manner of Dr. Stephens's letter is no better than its matter. Somebody is a panacea, and somebody else displays "opsomaniac tendencies towards calcium." These be hard sayings. As to anonymity, I think it would not be a bad thing, as rules go, if this were compulsory in little discussions of the present description. Only in that case we might have to scan the columns of our Hospital a trifle more narrowly for them. Yours, etc., J.

